# The Bruce effect revisited: is pregnancy termination in female rodents an adaptation to ensure breeding success after male turnover in low densities?

**DOI:** 10.1007/s00442-017-3904-6

**Published:** 2017-08-09

**Authors:** Jana A. Eccard, Melanie Dammhahn, Hannu Ylönen

**Affiliations:** 1 Animal Ecology, University of Potsdam, Maulbeerallee 1, 14469 Potsdam, Germany; 2 Biological and Environmental Sciences, University of Jyväskylä, Jyväskylä, Finland; 3 Konnevesi Research Station, University of Jyväskylä, Jyväskylä, Finland

**Keywords:** Breeding strategies, Dip test, Infanticide, *Myodes* voles, Sexual conflict, Sexual selection

## Abstract

Pregnancy termination after encountering a strange male, the Bruce effect, is regarded as a counterstrategy of female mammals towards anticipated infanticide. While confirmed in caged rodent pairs, no verification for the Bruce effect existed from experimental field populations of small rodents. We suggest that the effect may be adaptive for breeding rodent females only under specific conditions related to populations with cyclically fluctuating densities. We investigated the occurrence of delay in birth date after experimental turnover of the breeding male under different population composition in bank voles (*Myodes glareolus*) in large outdoor enclosures: one-male–multiple-females (*n* = 6 populations/18 females), multiple-males–multiple-females (*n* = 15/45), and single-male–single-female (MF treatment, *n* = 74/74). Most delays were observed in the MF treatment after turnover. Parallel we showed in a laboratory experiment (*n* = 205 females) that overwintered and primiparous females, the most abundant cohort during population lows in the increase phase of cyclic rodent populations, were more likely to delay births after turnover of the male than year-born and multiparous females. Taken together, our results suggest that the Bruce effect may be an adaptive breeding strategy for rodent females in cyclic populations specifically at low densities in the increase phase, when isolated, overwintered animals associate in MF pairs. During population lows infanticide risk and inbreeding risk may then be higher than during population highs, while also the fitness value of a litter in an increasing population is higher. Therefore, the Bruce effect may be adaptive for females during annual population lows in the increase phases, even at the costs of delaying reproduction.

**Electronic supplementary material:**

The online version of this article (doi:10.1007/s00442-017-3904-6) contains supplementary material, which is available to authorized users.

## Introduction

Sexual conflict is prevailing in most sexually reproducing species due to sexual dimorphism in gamete size, costs involved in mating and differential parental investment. Infanticide by non-parental males is a prominent example of the evolutionary conflict between the reproductive interests of males and females (Parker 1979, 2006) with females loosing reproductive investment, while males increase their chance of mating with the female. In mammals, infanticide has primarily evolved in group-living species, where reproduction is monopolised by a few dominant males (Lukas and Huchard 2014), and the care for dependent young prevents or delays females to engage in a subsequent reproductive attempt until current offspring are independent. Infanticide by non-parental males may, thus, have evolved to increase the infanticidal male’s chances to reproduce with the female by shortening the inter-birth interval (Hrdy 1979).

In turn, females evolved several counterstrategies to male infanticide (summarised in Lukas and Huchard 2014), including promiscuity to confuse paternity, direct attack of potential perpetrators (Ylönen and Horne 2002), avoidance of infanticidal individuals, territoriality, as well as early termination of pregnancy (reviewed in Ebensperger and Blumstein 2007), the latter can also be called the ‘anticipated infanticide avoidance hypothesis’. This cessation of pregnancy at early stages is known as pregnancy block or the Bruce effect (Bruce 1959, 1960; Milligan 1976) and might reduce the energetic investment into young that are under threat to be killed by an invading male (Hrdy 1979; Schwagmeyer 1979; Ebensperger 1998; Roberts et al. 2012). Delayed pregnancies after take-over of one-male groups by a new male are often considered as an indicator of the Bruce effect, and were observed in free-ranging primates (*Theropithecus gelada*: Roberts et al. 2012), rodents (*Marmota marmota*: Hackländer and Arnold 1999), and odd-toed ungulates (*Equus caballus*, Berger 1983). Female mice (*Mus musculus*) might block pregnancies before implantation (Bruce 1959), but many other mammals disrupt pregnancies at later stages (voles: Stehn and Jannett 1981; Heske and Nelson 1984; Heske 1987, rats: Marashi 2012). Although early and late pregnancy terminations may be caused by different physiological mechanisms, they are often also subsumed under a broader Bruce or Bruce-like effect; we follow this classification. Further adaptive explanations for the Bruce effect include pregnancy blocking if it increases genetic compatibility (Yamazaki et al. 1983, but see Rülicke et al. 2006).

Hitherto evidence for the Bruce effect in small rodents comes from captive conditions (e.g., house mice: Bruce 1959; Parkes and Bruce 1961; Yamazaki et al. 1983; Rülicke et al. 2006; voles: reviewed in Stehn and Jannett 1981; vole pairs in very small (<4 m^2^) enclosures: Heske and Nelson 1984; Heske 1987, Norway rats: Marashi 2012). Mainly due to the lack of conclusive field evidence, the adaptive value of this potential female counterstrategy to male infanticide remains elusive in small rodents (Heske and Nelson 1984). In wild populations, females often show ovulation scars in spring, similar to those from experimentally induced pregnancy interruptions (Mallory and Clulow 1977), but these scars may as well be a consequence of sterile matings, triggering or ‘priming’ reproduction (Westlin 1981).

Evidence from experimental population studies on small rodents is also ambiguous. In such studies, large grassland enclosures were stocked with mixed-sex vole populations in high to very high densities. Populations that underwent an experimental replacement of males were compared to socially stable controls. As a result, timing of the first litter was delayed in some females after turnover of males (*Microtus canicaudus*: de la Maza et al. 1999, *Microtus ochrogaster*: Mahady and Wolff 2002), a second litter was delayed (*Microtus oeconomus*: Andreassen and Gundersen 2006), or recruitment of offspring was delayed (Mahady and Wolff 2002; Andreassen and Gundersen 2006; Opperbeck et al. 2012). Using mean values of reproductive timing per population, infanticide and pregnancy termination could not be disentangled and the use of population mean values potentially blurred information on females’ individual decisions. Although the results were somehow similar among all studies, they were interpreted as either “no support” (de la Maza et al. 1999), “very little support” (Mahady and Wolff 2002), an “indication” for the occurrence of a Bruce effect (Andreassen and Gundersen 2006), or the Bruce effect was not considered in the interpretation of results (Opperbeck et al. 2012). Hence, field evidence for the occurrence of pregnancy termination as a potential female counterstrategy to male turnover in small rodent populations is equivocal. Some authors have, therefore, considered the Bruce effect in rodents a laboratory artefact (Wolff 2003), where a caged female cannot avoid the male. Here, we suggest that the captive conditions producing a Bruce effect in small rodents have not yet been compared to analogous field conditions, and that the Bruce effect may be adaptive and common in nature in fluctuating rodent populations, however, only under a limited range of social and reproductive conditions, which we aim to identify in this study.

In many rodent species, populations undergo annual density fluctuations associated with changes in age structure as well as fundamental aspects of the social and breeding system. For example, in striped mice (*Rhabdomys pumilio*), females breed solitarily in low density or in one-male groups in high density (Schradin and Pillay 2005). Similarly, prairie voles (*M. ochrogaster*) breed monogamously at low density, but polygynandrously at high density (Lucia et al. 2008; Streatfeild et al. 2011). Thus, considering variation in population density, sex ratio, or breeding system as aspects of the social environment appears to be crucial to assess the adaptive value of pregnancy termination. We suggest that the Bruce effect in rodents may be an adaptation in fluctuating populations to breeding in single-female–single-male breeding units at low densities in the increase phase, and to the associated high risk of inbreeding or infanticide. With spring litters having a high reproductive value in increasing seasonal populations, preventing infanticide or inbreeding by pregnancy termination may be highly adaptive for a rodent female in the increase phase of the cycle, even at the costs of delaying her reproduction.

**Fig. 1:**
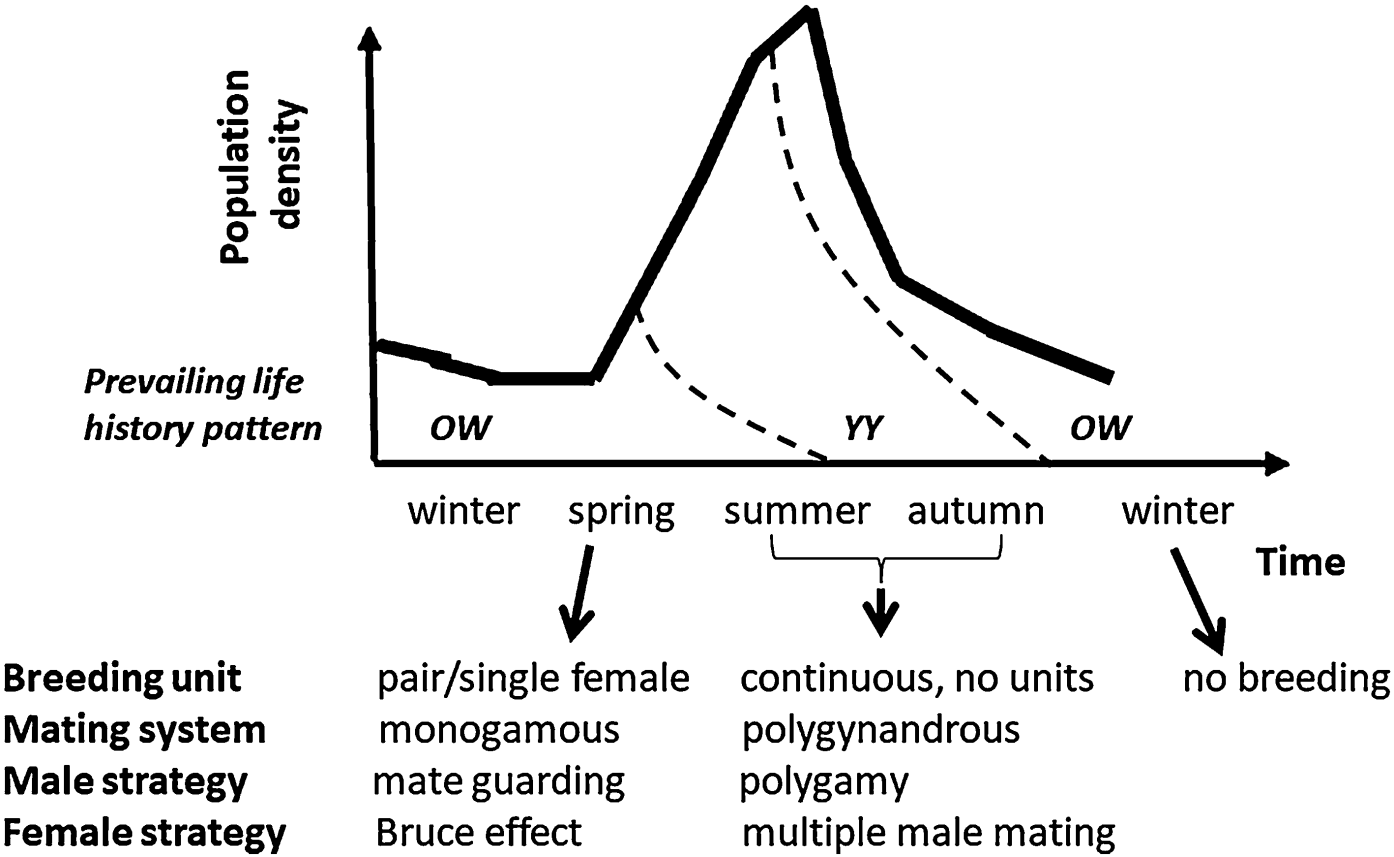
Counter-strategies of female rodents to infanticide risk or inbreeding risk adapted to the annual population density cycle and population structure (*OW*: overwintered, *YY*: yearborn, for maturation and reproduction of age classes see also Eccard and Herde 2013). In very low spring densities rodents may breed in pairs but change to polygynandrous breeding in high summer densities. Selection pressure on breeding behaviour may be highest during population bottlenecks in spring because of high fitness value of spring litters in increasing populations

We studied if a delay in birth date, likely due to the Bruce effect occurred in rodent populations under semi-natural conditions in different social environments, using bank voles (*Myodes glareolus*) as the study system. Bank voles have a polygynandrous breeding system when possible (Klemme et al. 2007, 2008) and show infanticidal behaviour of males (Ylönen and Horne 2002). Bank vole populations show annual density fluctuations with lows during winter and spring, and small social aggregations as well as large breeding groups were observed in the wild (Ylönen et al. 1988; Ylönen and Viitala 1991). Population highs build up during summer and start to decline in autumn and reach the lowest densities during subsequent spring (Fig. 1). They also show multiannual density fluctuation in part of their range (Yoccoz et al. 2001; Crespin et al. 2002) and during the population low or crash the densities reach very low values, which we expect to affect the breeding strategy of surviving individuals. Many small rodent genera have very similar population characteristics. The Bruce effect was shown in captivity (*Mus*: Bruce 1959, 1960; Milligan 1976; *Microtus*: e.g. Mallory and Clulow 1977; Stehn and Jannett 1981), we, therefore, assumed that bank voles were suitable for our study. To confirm that bank voles are able to delay births, and to confirm that the time interval of male turn-over chosen for our semi-natural study could potentially produce a delay of births, we conducted a side study on captive bank vole pairs which is also presented in this article.

In a series of experiments in semi-natural, large grassland enclosures we investigated the effect of social environment on the delay of birth dates. We created different social population structures differing pronouncedly in the number of possible mating partners. In each experiment, we compared the timing of births in stable population where we did not expect the Bruce effect, with the timing of birth in populations with turnover of males, where the Bruce effect can be expected. Populations consisted either of a single female with a single male (MF), multiple females with a single male (MFFF), or multiple females with several males (MMMFFF). We predicted,

1. If pregnancy termination after male turnover is adaptive only for a female breeding in pair constellation, i.e., with one male in a low density phase, but not for promiscuously or harem breeding females in higher densities, a delay in birth date should be more common in the MF experiment than in any of the group experiments [rate of late births (MF > (MFFF and MMMFFF)]. As discussed above, earlier studies on the Bruce effect in experimental populations were always conducted in multi-male–multi-female populations (de la Maza et al. 1999; Mahady and Wolff 2002; Andreassen and Gundersen 2006; Opperbeck et al. 2012), which may explain equivocal results.
2. As an alternative hypothesis, pregnancy termination may be an alternative strategy to paternity confusion avoiding infanticide or inbreeding. If so, delay in birth dates should be more common in the single-male populations without additional males to mate with, than in a multi-male population. Many rodents breed promiscuitively (Eccard and Wolf 2009; Klemme et al. 2007). To test this hypothesis, we compared multi-female populations and expected a higher rate of late births in single-male versus multi-male conditions (MFFF > MMMFFF). In Southern African vlei rats (*Otomys irroratus*) originating from “polygynous populations” females had higher rates of pregnancy termination in caged pair conditions than females from “promiscuous populations” (Pillay and Kinahan 2009), supporting our hypothesis.
3. If absolute animal density affects the Bruce effect (as proposed in the lab artefact discussion, Wolff 2003), delayed births should be more common at higher compared to lower densities (MMMFFF > MFFF > MF) in same sized enclosures. Moreover, for MF experiments, late births should be more common in smaller (higher density) than in larger (lower density) enclosures (MF smaller > MF larger enclosures).

To investigate the evolutionary significance of the Bruce effect in annual population structure and dynamic, we compared rates of delayed births in different age cohorts of females in the aforementioned parallel study in captive bank voles and in some of the multi-female populations. In natural populations, annual density fluctuations go along with changes of demographic structure (Eccard and Herde 2013). Low densities occur annually in spring (Fig. 1), when the majority of individuals are overwintered (OW) and first time breeders (nulliparous (n), here referred to OW-n). The first generation of offspring born in the same year (young of year, YY), breeds for the first time (YY-n) at an intermediate density, depending on the number of founder females. Later in the breeding season, all breeders are experienced, established breeders [parous (p)] and can be of both age cohorts (OW-p and YY-p). Towards the end of the year, densities are high and breeding territories occupied, than young YY females cannot enter the breeding population and their maturation is suppressed (Prevot-Julliard et al. 1999). Accordingly, if the Bruce effect is adaptive in low population densities, we expected higher rates of delayed births in the cohorts most common during population lows.

4. Specifically, we predict higher rates of late births in overwintered, first time breeders compared to year-borne, experienced breeders [OW-n > (YY-p and OW-p)]. The combination YY-n, which occurs at intermediate densities, may be intermediate also in the rate of delayed births. There are indications that females of different cohorts are differentially likely to show the Bruce effect: first time breeders more likely interrupted a pregnancy than experienced breeders (Stehn and Jannett 1981; Clulow et al. 1982, but see Chipman and Fox 1966); and younger females more likely than older females (Clulow and Langford 1971; Heske 1987).

Combining both laboratory and field data, we aimed to identify social environmental and demographic conditions under which the Bruce effect occurs, and relate these to natural fluctuations of population density and demography to understand whether and when the Bruce effect could be an adaptive breeding strategy for female rodents in low density populations in the population increase phase (Fig. 1).

## Materials and methods

The study was conducted on colony-bred bank vole females (*M. glareolus*) and encompasses four experiments. In 1999–2001 in Konnevesi, Central Finland, we conducted two field experiments in multi-female groups with different number of males. In 2014–2015, we conducted a third field experiment near Potsdam, Eastern Germany, on populations of a single breeding pair. Enclosure sizes, colony origin of voles, time schedules and turnover treatments of the experiments were corresponding (Table 1). Experiments differed in location, year, marking method of voles and composition of populations. The fourth part of the study was a breeding experiment on captive voles conducted in Konnevesi in 1999–2000, where we used the same turnover treatments and breeding schedules as in all field experiments. Voles at both facilities were bred in standard mouse cages at 18–22 °C and fed on lab chow with water ad libitum. Voles were kept at 14–16-h daylight during summer breeding season and 8-h daylight during non-breeding season in winter. Experiments were integrated into breeding routines of voles in colony and enclosures.

**Table 1:** Experimental population experiments on pregnancy termination in bank voles in large outdoor enclosures with different population compositions: multi-male–multi-female (MMMFFF), single-male–multi-female (MFFF), and an isolated breeding pair (MF), where each M (and each F) resembles one male (one female, respectively) per population

	Composition of population					
	MMMFFF		MFFF		MF	
Location	Finland		Finland		Germany	
Year	1999		1999 + 2000		2014 + 2015	
Enclosure size large/small [m^2^]	2500/–		2500/–		2500/255	
Vole density [ind./ha] large/small	24/–		16/–		8/88	
Females						
Overwintered, nulliparous (OW-n)	2		12		–	
Overwintered, parous (OW-p)	6		6		–	
Yearborn, nulliparous (YY-n)	4		25		74	
Yearborn, parous (YY-n)	6		2		–	
Sample sizes						
Turnover treatment	Replace	Return	Replace	Return	Replace	Return
No. of experimental populations	3	3	10	5	38	36
No. of recaptured females	8	9	23	14	27	18
No. of births of litters	8	7	17	12	21	14
No. of births with complete retrieval of experimental males from field	8	7	17	12	11	10
No. of births from nulliparous mothers with complete retrieval	3	1	14	10	11	10

After 1 week original males were removed and either replaced by a different male or returned (turnover treatments). Population replicates were conducted in rounds with simultaneous returned and replaced treatments. Original data are available in the data ESM appendix

### Study species and experimental animals

Bank vole females were offspring (F1 or F2) of wild caught animals and were either first time breeders (nulliparous, n) or experienced breeders (parous, p). They were either born in the previous autumn (OW) or the same season (YY). Nulliparous, overwintered females (OW-n) had an age of 5–7 months and a body mass of 18–25 g. Nulliparous YY females (YY-n) were 1–2 months old and weighed 10–15 g at the start of the experiment. Parous OW (OW-p) and YY females (YY-p) were 7–12 and 5–8 months old, respectively, and weighed between 18 and 30 g. Males were 5–12 months old and had a body mass of 18–30 g. Males were captured directly from the wild or had been experienced and successful breeders in the laboratory. Sample sizes of the female cohorts are given under the respective experiments.

The schedule of male turnover treatments was the same for all experiments, both experimental populations and captive pairs. Male turnover was conducted in weekly intervals and births were recorded as experimental days. After release to the enclosure/breeding cage (experimental day 1) voles were kept together for seven days. On experimental day 7, original males were removed and either returned to the enclosure/breeding cage (returned male treatment) or replaced by different individuals (replaced male treatment). Animals were kept together for another 7 days (experimental days 7–14). After removal from enclosure/breeding cage at day 14, females were kept in separate cages and inspected daily to document occurrence of pregnancy and birth dates. With a bank vole pregnancy lasting 20 ± 2 (mean ± SD) days (Bujalska 1983), we assumed that litters born *early* during experimental days 18–25 were conceived during days 1–7. Litters born late during experimental days 26–34 were conceived during days 7–14 and could be sired either by the returned original male or the replacement male, depending on turnover treatment.

#### Three field experiments: replacement of male(s) in different population compositions

We investigated the occurrence of delayed births in three experiments with two parallel male turnover treatments within each experiment. Experiments corresponded in the species used, habitat type in enclosures, size of enclosures, type and number of traps, trap control intervals, and schedule of male turnover (Table 1). Experimental populations were kept in large (50 m × 50 m) grassland enclosures or, in the MF experiment, we additionally included a set of smaller (15 m × 15 m) enclosures, (Table 1). Enclosures had a permanent trapping grid of Ugglan multiple capture life traps (Grahn AB, Sweden).

Experiments differed by their population composition: (1) single-male–single-female population (MF) with one male and one female per enclosure (*n* = 74 populations, 74 females), (2) single-male–multiple-female populations (MFFF) with one male and three females per enclosure (*n* = 14 populations, 42 females), (3) multi-male–multi-female populations (MMMFFF) with thee males and three females per enclosure (*n* = 6 populations, 18 females). Experiments also differed by the combination of female age and reproductive history, location, animal marking method and years (Table 1). Within each experiment, we conducted several simultaneous replicates of the returned male and replaced male treatments.

In the multi-female experiments, we released three females per population to an enclosure, which allows all of them to breed (Eccard et al. 2011). According to seasonal availability of different cohorts (Fig. 1), experimental populations consisted of females of different ages or reproductive history (Table 1), always distributed equally across the populations.

Voles were individually marked and released to the centre of the enclosure on experimental day 1. Live-trapping was conducted after 1 week for one night, after which females were returned to the original enclosure, while males were either replaced with different individuals or returned. In total, we released 137 vole females to the enclosures and recaptured 99 females of which 79 were gravid. After excluding those replicates where recapture of males was incomplete, we analysed the timing of birth for 65 gravid females (Table 1). We repeated analyses of timing of birth for a data set restricted to nulliparous females (*n* = 49 females, 4 MMMFFF, 24 MFFF, 21 MF) where we had to pool data of multi-female populations (4 + 24 = 28 females).

Vole densities in the large enclosures (Table 1) corresponded to densities in the increasing phase, whereas densities in the small enclosure represented the peak phase of the population cycle (Ylönen et al. 1988; Eccard et al. 2011), allowing us to investigate the effect of population composition within large enclosures (among experiments) and of densities within MF experiments (between large and small enclosures).

#### Pairing experiment on captive bank voles: effects of female cohort and replacement of the pair male on timing of pregnancies (MF)

Pairings were conducted in spring 2000 as part of the breeding routine in the bank vole colony. Females of the replaced male treatment were transferred to a clean cage or the males’ cage and paired to a different male during experimental days 7–14. Females of the returned male treatment were transferred to a clean cage and paired again to the original male during day 7–14. In total, we monitored pairings of 204 females of three different age and parity cohorts (*N* = 113 OW-n females, *N* = 59 YY-n females and *N* = 32 YY-p females) with a male.

### Statistical analyses

We obtained binary response variables of the occurrence of birth versus no birth; and of early versus late births, respectively. Assuming that the Bruce effect is restricted to specific compositions of the population, we expected interactive effects of male turnover treatment with population composition (field experiments) on the timing of births. Assuming that the Bruce effect is restricted to specific age cohorts of females, we expected interactive effects of male turnover treatment with female cohort (laboratory experiment) on the timing of births. We, therefore, modelled these interactive effects both on the probability of females giving birth, and the probability of late births in separate generalised linear models (GLMs) with binomial error distribution. If the statistical model indicated a marginal support (*p* < 0.1) for any of the specified interactions among two factors, we subsequently investigated simple effects of one factor within levels of the other factor using a Chi^2^-test, or in case of low sample size using Fisher’s exact test, as post hoc tests for an association.

Since in previous experiments on the Bruce effect in other rodent species (e.g., Clulow et al. 1982) not all but a fraction of females terminated pregnancies after male replacement, we expected a bimodal distribution of birth dates for the replaced male treatments, including a first peak of early births around day 20 and a second peak of late births around day 27. For the returned male treatment, we expected a unimodal distribution, peaking at early births around experimental day 20. To investigate modality in birth dates we used the Hartigans’ Dip test for unimodality (Hartigan and Hartigan 1985) run with the R-package ‘dip test’ (Maechler 2013). This test detects deviations from unimodality (i.e., *p* < 0.05 indicates non-unimodality). Upon detection of a deviation, we inspected the histograms of birth day (experimental day) frequencies and report the location of modes.

Nulliparous bank vole females in laboratory colonies not always conceived after being paired to a male, while once females started breeding they easily continue to breed (J.A.E. own observations). Hence, offering an additional male in the replaced male treatments may simply increase the chance of mating with a compatible partner. Since we were not able to distinguish between replaced pregnancies and additional pregnancies, we also analysed the pregnancy rate (i.e., the occurrence of pregnancy) from the experiments. We assumed that a combined increase of late pregnancy rate AND of overall pregnancy rates would indicate the occurrence of additional pregnancies, while an increase of late pregnancies without higher overall pregnancy rates would indicated a replacement of original pregnancies with the respective new male. A termination of pregnancy without a continuation of breeding, we assume to be highly unlikely, since once short lived rodent females get into breeding condition, they easily continue to breed.

The three females within an experimental MFFF or MMMFFF population may not be seen as completely independent samples, we, therefore, used generalised linear mixed model (GLMM) including population as a random factor (Zuur et al. 2009) in the analysis of multiple-female experiments (Hypothesis 2 and 4).

Statistical analyses were performed with R 3.1.0 (R-Development-Core-Team 2013) using R-studio (RStudio Team 2015) and R-commander (Rcmdr, Fox 2005), and the packages ‘lme4’ (version 1.1-6, Bates et al. 2014) and ‘car’ (version 2.0-20, Fox and Weisberg 2011). Original data can be found in the online material (ESM Appendix 1).

## Results

**Fig. 2:**
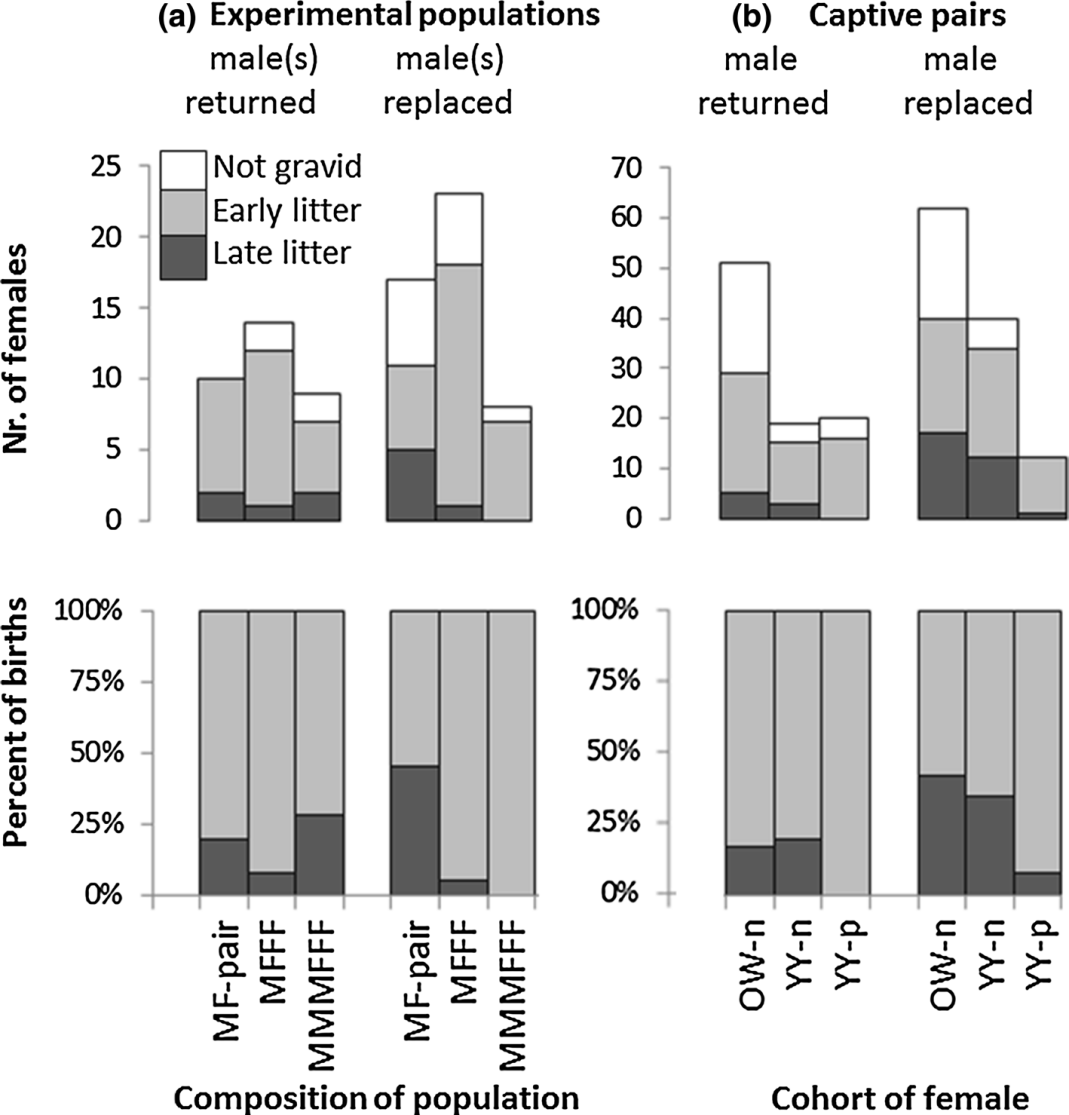
Laboratory and field experiments on conception and pregnancy termination (Bruce effect) in bank voles (*Myodes glareolus*) after turnover of breeding males (breeding male replaced, or returned (control) after capture). **a** Different population composition of males (M) and females (F) in separate large outdoor enclosures [*n* = 84 females, multi-male–multi-female (MMMFFF), single-male–multi-female (MFFF), or isolated male–female (MF)]. **b** Laboratory experiment with caged male–female pairs (*n* = 204), females differed by age (*OW* overwintered, *YY* yearborn) and reproductive history (*-n* nulliparous, without birth prior to the first pairing, *-p* parous: had reproduced before the trial)

In the field experiments, 65 births were observed in 81 recaptured females (80% pregnancy rate). A total of 11 litters (17% of the births) were born late. In the full model, there was a tendency for an interaction effect of population composition with male turnover treatment on the probability of late births (turnover treatment: *Χ* ^2^ = 0.0, *df* = 1, *p* = 0.93, composition: *Χ* ^2^ = 6.2, *df* = 2, *p* = 0.045, interaction *Χ* ^2^ = 4.8, *p* = 0.093, Fig. 2, Table 2, for post hoc tests see respective H1 and H2 below).

**Table 2:** Eccard et al. Oecologia, Bruce effect

Hypothesis, data base (sample size: early/late births, Model type)	Explanatory variable	Estimate	SE of estimate	*Z* value	*p*
H1: Pair vs multiple breeders, population experiments (*n* = 57/11, GLM)	(Intercept)	1.7	0.6	2.7	0.008
	Turnover	1.5	1.2	1.3	0.210
	Population composition	0.3	1.0	0.3	0.776
	*Interaction*	*2.2*	*1.6*	*1.7*	*0.082*
H4: Cohort effects, caged pairs (*n* = 138/38, GLM)	(Intercept)	1.5	0.4	3.6	<0.001
	**Turnover**	**1.1**	**0.5**	**2.5**	**0.011**
	YY-n:OW-n	0.1	0.4	0.5	0.652
	**YY-p:OW-n**	**2.5**	**1.1**	**2.3**	**0.020**
	**YY-p:YY-n**	**2.3**	**1.1**	**1.1**	**0.035**
H2: Single vs multiple male, population experiments female groups (*n* = 40/4, GLMM)	(Intercept)	0.4	0.7	2.1	0.031
	Turnover	0.7	0.8	0.9	0.346
	No. of males	0.1	1.0	0.1	0.897
	Interaction	0.7	1.4	0.5	0.621
H4: Cohort effects, population experiments female groups (*n* = 40/4, GLMM)	(Intercept)	9.9	10.0	1.0	0.327
	Turnover	2.1	9.3	0.1	0.920
	Age of females (OW:YY)	2.5	7.6	0.3	0.739
	Reproductive history of females (n:p)	4.1	7.1	0.6	0.563

After 1 week original males were removed, turnover treatments included replacing or returning the original male. Population experiments included pairs (MF), single-male groups (MFFF) and multi-male–multi-female (MMMFFF) groups. Females in the laboratory experiment and females in the field experiment with female groups differed in cohort (age and breeding experience combined, *OW-n* over-wintered nulliparous, *YY-n* year-born nulliparous, *YY-p* year-born parous). Significant effects are indicated in bold, tendencies in italics

In the experiment on captive pairs a total of 146 litters were born to 204 females (72% pregnancy proportion), of which 26% (38) were born late. Both male turnover treatment and cohort explained late litters rates, without interaction (turnover treatment: *Χ* ^2^ = 7.1, *df* = 1, *p* = 0.008, cohort: *Χ* ^2^ = 10.1, *df* = 2, *p* = 0.008, interaction: *Χ* ^2^ = 1.0, *p* = 0.56, Fig. 2; Table 2, discussed at respective hypotheses H1 and H4 below).

### H1: The Bruce effect in single-male–single-female breeders [proportion of late litters: MF > MFFF and MF > MMMFFF)]

**Fig. 3:**
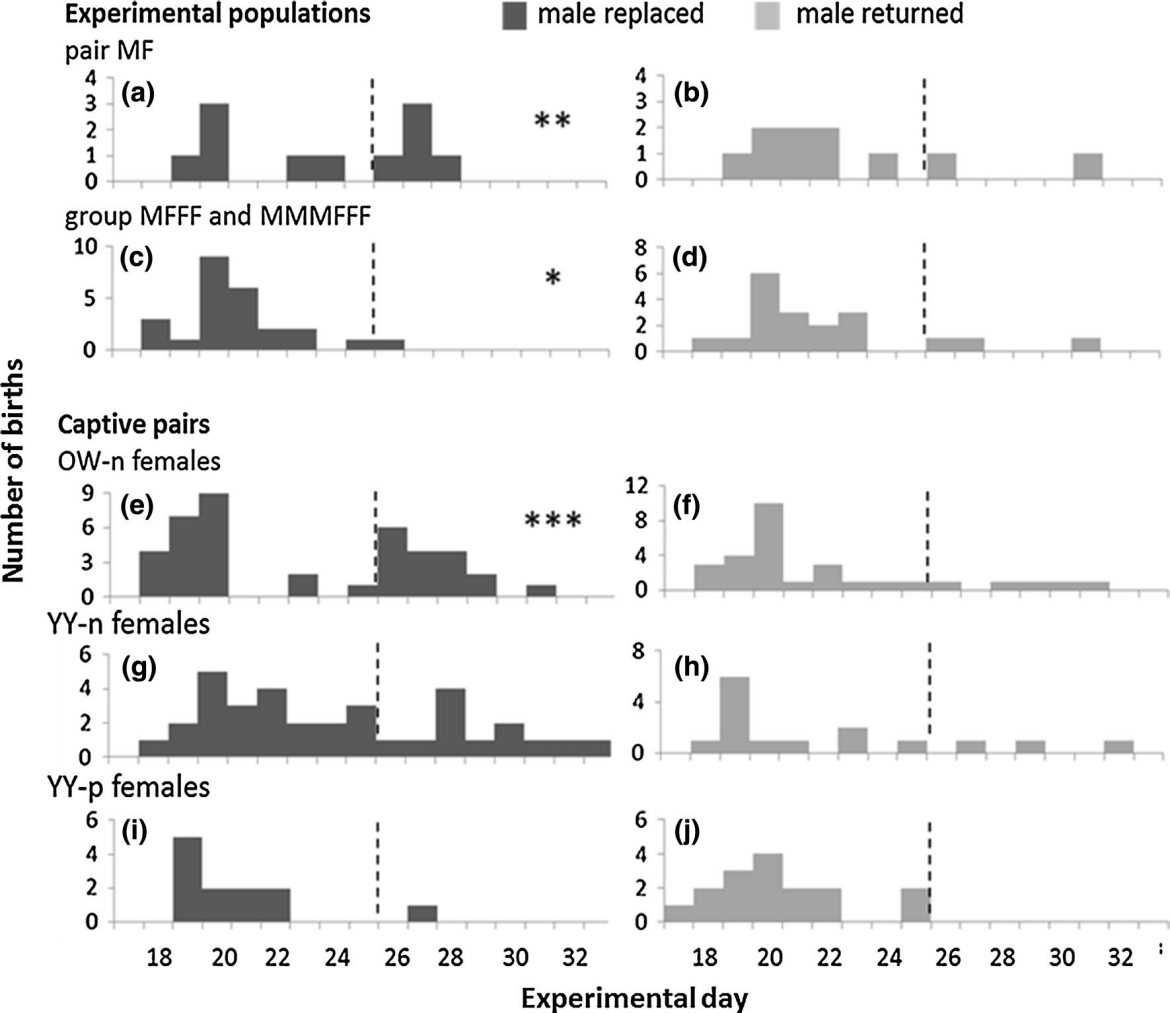
Distribution of birth dates of bank vole litters after male turnover (*black*) or male return (control, *grey*). Experiments with different population compositions (**a**–**d**), and caged pairs (**e**–**j**) with different females’ ages (*OW* overwintered, *YY* yearborn) and female reproductive history (*-n* nulliparous, *-p* parous). *Hatched lines* divide early (conceived before turnover date) and late births (conceived after turnover date, possibly due to the Bruce effect). *Stars* indicated deviations from unimodal distributions (Hartigans’ dip test (Hartigan and Hartigan 1985) with *p < 0.05, **p < 0.01, ***p < 0.001, with **a** and **e** indicating distributions with modes at both early and late birth intervals, while in **c** early birth was divided in two modes

After a turnover of the breeding male, females in MF conditions had a higher proportion of late litters compared to females in multi-female conditions (simple-effect tests within treatment levels: within the replaced male treatment, the proportion of late litters was associated to population composition (Table 3): MF females produced a higher proportion of late litters (45%, 5 out of 11) than MFFF females (6%, 1/17, Fishers exact: *p* = 0.018) and tended to produce a higher proportion of late litters than MMMFFF females (0%, 0/7, *p* = 0.10). MFFF and MMMFFF females did not differ in the proportion of late litters (6 and 0%, *p* = 1.00). In the data set restricted to nulliparous females these results were confirmed: after male replacement the proportion of late litters in MF females (45%, 5/11) was higher than from (MFFF + MMMFFF) females (6%, 2/28, *p* = 0.018; all other within-factor comparisons *p* > 0.36). Dip tests indicated non-unimodal distribution of births in replaced male treatments (Fig. 3a, c), but while there were two modes (early births and late births) in the MF treatment (3c), there were two distinct modes of early births in the group treatments.

**Table 3:** Post hoc tests within factor levels on the proportion of early to late births born in different male turnover treatments and population compositions in three field experiments on pregnancy termination in bank voles

Within factor	Within-factor levels	Among factor levels	No. of early births	No. of late births	Fischer’s exact *p*
Male turnover treatment	Returned male	MMMFFF	5	2	0.58
		MFFF	11	1	
		MF	8	2	
	Replaced male	**MMMFFF**	**7**	**0**	**0.023**
		**MFFF**	**17**	**1**	
		**MF**	**6**	**5**	
Composition of population	MMMFFF	Returned males	5	2	1.00
		Replaced males	7	0	
	MFFF	Returned male	11	1	0.46
		Replaced male	17	1	
	MF	Returned male	8	2	0.36
		Replaced male	6	5	

After 1 week original males were removed, turnover treatments included replacing or returning the breeding male. Populations were either composed of multi-males–multi-females (MMMFFF), single-male–multi-females (MFFF), or an isolated male–female breeding pair (MF). Significant effects are indicated in bold

Within the returned male treatment in the full data set the proportion of late births (19%, 5/29) was not associated to population composition (*p* = 0.58). Within none of the population compositions, the proportion of late litters was associated with male turnover treatments (returned and replaced treatments in MF pairs: 20 and 45%, in MFFF: 8 and 6%, in MMMFFF: 0 and 29%, respective absolute numbers in Table 2, all *p* > 0.36). Dip tests in the returned treatment (Fig. 3b, d) did not support other than one (early birth) mode.

In the experiment on captive pairs, male replacement resulted in a higher probability of late births (30 late out of 86 births = 35%) compared to if the male was returned (8/60 = 13%, Fig. 2).

### H2: The Bruce effect as an alternative to paternity confusion (proportion of late births: MFFF > MMMFFF)

Between the multi-female group experiments, there was no effect of single male versus multiple males on the proportion of late births (4 late out of 44 total, 9%, Figs. 2 and 3), and we found no interactive effect of male turnover and population composition (GLMM, number of males: *Χ* ^2^ = 0.0, *df* = 1, *p* = 0.92; male turnover treatment; *Χ* ^2^ = 0.0, *df* = 1, *p* = 0.83, female age: *Χ* ^2^ = 0.1, *df* = 1, *p* = 0.74; female breeding status: *Χ* ^2^ = 0.3, *df* = 1, *p* = 0.56, Table 2).

#### H3: Absolute animal density affects the Bruce effect (proportion of late births; in large enclosures: MMMFFF > MFFF > MF) and (MF small > MF large)

Density did not affect the proportion of late births (*Χ* ^2^ = 1.7, *df* = 2, *p* = 0.42, *n* = 54 females in large enclosures). Within the MF experiment we had used two enclosure sizes (5 births from small/5 from large in replace, 7/4 in return treatment). Pregnancy rate was 75% in both enclosure sizes, and the proportion of late births was 42% in smaller enclosures and 22% in larger enclosures. An interaction of enclosure size and turnover treatment did not explain the probability of late births to occur (interaction: *Χ* ^2^ < 0.4, *p* > 0.50).

### H4: Cohort effects on proportion of late births (OW-n > YY-n > YY-p)

In the pairing experiment on captive bank voles, the proportion of late births was affected by both male turnover treatment and female cohort (Fig. 2; Table 2). OW-n and YY-n females did not differ in the proportion of late births (32 and 31%, *Χ* ^2^ = 0.0, *df* = 1, *p* = 1.00), but YY-p females had a lower proportion of late births than the other cohorts (4%, *Χ* ^2^ = 7.3, *p* = 0.007 and *Χ* ^2^ = 6.4, *p* = 0.012). Although we could not detect a cohort-specific treatment effect in the statistical model (non-significant interaction effect), we found that the distribution of birth dates deviated from unimodality only for OW-n females in the replaced male treatment. (*D* = 0.11, *p* < 0.001) with a distinct additional second peak from late births at day 26, and a first peak at day 19 (Fig. 3e) There was no indication of deviation from unimodality in this cohort in the male returned treatment, and in no other combinations of cohort and treatment (all *D* < 0.09, all *p* > 0.25, Fig. 3).

In the field experiments with groups of females (MMMFFF and MFFF), we had also used different cohorts of females. The proportion of late pregnancies in multi-female experiments was very small (9%) and not depend on female age or cohorts (Table 2, please note that the small sample size did not allow testing effects of male number (H2) and female cohorts (H4) in the same model. We therefore ran separate models for the two hypotheses).

### Additional late pregnancies or replacement of early pregnancies?

With an imbalanced distribution of the response variable (gravid or not) among population compositions and the occurrence of empty cells, it was impossible to run a binary model across all groups. Using post hoc tests within levels, we found no simple effects of population composition within turnover treatments on pregnancy rate, and we found no simple effects of turnover treatments within population compositions on pregnancy rates (all Fisher’s exact tests, *p* > 0.49).

In the multi-female experiments (MFFF vs MMMFFF), 44 litters were born to 54 recaptured females (81%). Neither male numbers (*Χ* ^2^ = 0.9, *df* = 1, *p* = 0.40) nor male turnover (*Χ* ^2^ = 0.2, *df* = 1, *p* = 0.64) explained the rates of pregnancy in these experiments. Parous females had higher pregnancy rates (16/17) than nulliparous females (28/37, *Χ* ^2^ = 3.9, *df* = 1, *p* = 0.048) and year-born females tended to have higher pregnancy rates (28/33) than overwintered females (15/20, one female with unknown age, *Χ* ^2^ = 2.7, *df* = 1, *p* = 0.099, Table 4).

**Table 4:** Pregnancy rates in multiple female groups of bank voles: results of generalised linear models (GLM) and mixed effect models (GLMM) with binomial error distribution and probit link function of the variables “probability of pregnancy”

Data base (sample size: pregnant/non pregnant, model type)	Explanatory variable	Estimate	SE of estimate	*Z* value	*p*
Field experiment with female groups (*n* = 44/10, GLMM)	(Intercept)	0.2	1.3	0.1	0.898
	Turnover	0.4	0.1	0.3	0.772
	No. males	1.0	0.1	0.7	0.480
	Interaction	0.1	2.0	0.1	0.955
	**Reproductive history (n:p)**	**2.8**	**1.4**	**2.0**	**0.045**
	Age (OW:YY)	1.4	1.0	1.4	0.158
Caged pairs (*n* = 146/62, GLM)	(Intercept)	0.2	0.3	0.8	0.450
	Turnover	0.5	0.3	1.5	0.148
	**OW-n:YY-n**	−**1.1**	**0.4**	**2.7**	**0.006**
	**OW-n:YY-p**	−**1.6**	**0.6**	**2.8**	**0.006**
	YY-n:YY-p	0.5	0.7	0.8	0.437

After 1 week original males were removed, turnover treatments included replacing or returning the breeding male. Population composition in the field experiments included single-male groups (MFFF) and multi-male–multi-female (MMMFFF) groups. Females in the laboratory experiment and females in the field experiment differed in age (*OW* overwintered, *YY* yearborn) and reproductive history (*n* nulliparous, *p* parous). Significant effects are indicated in bold

In the pairing experiment on captive voles, the replacement of the original male did not add additional pregnancies (*Χ* ^2^ = 2.1, *df* = 1, *p* = 0.15). The probability of pregnancy differed among female age cohorts though (*Χ* ^2^ = 14.9, *df* = 2, *p* < 0.001). Pregnancy rates were higher in both YY-n and YY-p (83 and 88%, *Χ* ^2^ test: *Χ* ^2^ = 0.1, *df* = 1, *p* = 0.80) as compared to OW-n females (61%, *Χ* ^2^ = 7.7, *p* = 0.006 and *Χ* ^2^ = 6.72, *p* = 0.01). We found no interaction of cohort and male turnover treatment (Table 4).

## Discussion

Breeding in MF pairs increased the proportion of late births after turnover of the male, compared to breeding in multi-female groups (supporting H1). Here we report first experimental evidence for pregnancy termination of female rodents in field populations after replacement of breeding male(s) by unfamiliar male(s). We conclude that pregnancy termination may be an adaptive behaviour to the specific, social condition of a single-female breeding with a single male, a condition which in small mammals in nature can occur only at low population densities in the increase phase of the annual population cycle. We suggest that the Bruce effect in a small mammal is not a laboratory artefact (as suggested by Wolff 2003), but represents a flexible adaptive response to male turnover in breeding pairs (Fig. 1).

Results from both captive pair and field experiments support the occurrence of late births after turnover; but we found no support for an increase of pregnancy rates. Although in the field experiment the overall statistical model of pregnancy rates indicated a tendency for an interaction of male turnover and population composition (Table 2), this interaction was not supported by simple effects post hoc tests. We, therefore, conclude that observed late births after male turnover were mainly a product of pregnancy termination rather than a significant proportion of additional pregnancies.

Breeding in single-female–single-male units at low population densities in the population increase phase may be a common feature of the social system of many rodent species with annual and multi-annual population density fluctuations. Some rodent species were observed to breed in isolated pairs at low densities but in large groups at high densities (mice: Schradin and Pillay 2005, voles: Lucia et al. 2008). Genetic monogamy was reported from low densities of voles, but genetic polyandry from high density (Streatfeild et al. 2011). Thus, depending on habitat saturation with breeding territories breeding systems of many rodent species are flexible.

For bank voles, a great variability in breeding systems has been reported so far. It includes female territoriality and breeding suppression above population density thresholds (Bujalska and Grüm 1989; Eccard and Ylönen 2001), loss of density dependence at very high densities (Eccard et al. 2011), high density group breeding (Ylönen et al. 1988) and small wintering aggregations of siblings (Ylönen and Viitala 1991). Social organisation at very low spring densities, and residence time of males at female nests has, to our knowledge, not been investigated in this species.

The adaptive value of pregnancy termination during a population bottleneck may also depend on the relatedness of the overwintered female to the breeding male. Bank voles overwinter in small aggregations of siblings and probably start breeding in spring with a locally available male sibling. An intruding unknown male may thus represent an adaptive opportunity for outbreeding. In mice, the chance for outbreeding increased the proportion of pregnancy blocks (Yamazaki et al. 1983, but see Rülicke et al. 2006). It remains to be tested experimentally whether this opportunity outweighs the costs for the female of delaying reproduction in cyclic populations.

Infanticide risk my males at low population density may differ from that at high population density. Paternity assurance for the breeding male is high in a pair-breeding condition. Consequently, after a turnover of the breeding male, a female’s litter may be at greater infanticide risk (Ylönen and Horne 2002) than in high population density. Further, in rodents with altricial young, close guarding and attending of the female by the new male increases the risk of the litter being found by the intruder. Thus, behaviour of a male which breeds with a single female may pose a greater infanticide risk than behaviour of a male breeding with multiple females. The Bruce effect may therefore function as a counterstrategy to infanticide in single-male–single-female breeding situations.

We found no support for the Bruce effect acting as an alternative to paternity confusion (H2) to counteract infanticide in groups of females, independent of single or m multiple breeding males late births were rare. In many other mammals with a group structure of monopolisable females (a harem), females are threatened to lose their offspring through infanticide after turnover of the dominant male (Lukas and Huchard 2014). Different from the Bruce effect observed in one-male groups in primates (Hrdy 1979; Roberts et al. 2012) or horses (Berger 1983), female rodents in one male groups seem not to have to interrupt pregnancies after male turnover.

Possibly, the incidence of the Bruce effect is also affected by male mating behaviour. Male mate-guarding intensity and frequency of sexual harassment by the males (Clutton-Brock and Parker 1995) is potentially lower for a female when alternative receptive females are available for the male (Parker 1974; Mathews 2002). Males with alternative breeding opportunities may be less insisting and can probably be avoided by the female, expelled from the territory, or aggressively evicted from the nest site (Koskela et al. 1997; Ylönen and Horne 2002), or the nest site can be hidden (Ebensperger 1998). According to socio-ecological theory, the distribution of risks and resources determines female distribution in space and time, whereas males adjust their spacing pattern to the distribution of receptive females (Clutton-Brock 1989; Altmann 1990). These patterns have been demonstrated experimentally, for example, for female grey mouse lemurs (*Microcebus murinus*: Dammhahn and Kappeler 2009) and male and female grey-sided voles (*Clethrionomys rufocanus*: Ims 1988).

The proportion of late births did not increase with absolute density (refuting H3). We found delayed births both in the highest densities (pairs in small cages) and the lowest densities (pairs in large enclosures), while a doubling or tripling of density in the large enclosures did not increase the incidence of pregnancy termination. Further, a 10-fold increase between larger and smaller enclosures within the MF experiment did not conclusively affect the occurrence of late births, although other processes like spacing behaviour, breeding suppression, and physiology can be affected by density (e.g., Eccard et al. 2011). These results suggest that female reproductive strategies are based rather on social cues like population structure or male sexual behaviour specific to pair breeding, than on density cues alone.

In house mice kept in groups (on very limited space), the incidence of pregnancy termination was not augmented by the number of males but reduced by the number of females (Bruce 1963), supporting our H1 and H3. Bruce discussed hormonal mechanisms and maladaptive effects of crowding, but her findings can also be interpreted as an adaptive flexibility of female reproductive behaviour to population composition and behaviour of the male, rather than being directly density related.

We found female cohort effects on the timing of births (supporting H4) in the breeding experiment on captive voles. Overwintered females without breeding experience (OW-n) had higher proportions of late births (Figs. 2, 3) than experienced young-of-the-year breeders. Female cohorts also differed in pregnancy rates, with YY females having higher pregnancy rates than OW females, and parous females having higher pregnancy rates than nulliparous females (Fig. 2). Overall, these results confirm earlier findings: in many rodent species, females breeding for the first time were more likely to interrupt their pregnancy than parous females after male turnover (Stehn and Jannett 1981; Clulow et al. 1982, but see Chipman and Fox 1966). Moreover, younger females are more likely to interrupt a pregnancy than older females (Clulow and Langford 1971; Heske 1987). In annually fluctuating rodent populations overwintered and first time breeding females are the prevailing cohort during the lowest annual population density in the population increase phase in spring (Fig. 1). Observations of ovulation scars in female voles captured in spring (Mallory and Clulow 1977) may further support our hypothesis that pregnancy termination may be of seasonal importance and restricted to specific reproductive strategies of low density spring populations, consisting of mainly OW-n females (Fig. 1). With age structure closely related to annual density cycles, our results suggest that females might adjust their counterstrategies flexibly infanticide risk by males, which varies according to the prevailing social environmental conditions (Fig. 1).

## Conclusions

Here, we provide first indication for delayed breeding after male turnover in experimental field populations of rodents, suggesting that the Bruce effect is not a laboratory artefact. The adaptive value of costly female counterstrategies in sexual conflicts probably depends on the social environment of the breeding female, and thus varies with population density and season. The Bruce effect in rodents may be an adaptation in fluctuating populations to breeding in single-female–single-male breeding units at low densities in the increase phase of the annual population cycle, and to associated increased risks of inbreeding or infanticide. With spring litters having a high reproductive value in increasing seasonal populations, pregnancy termination to prevent infanticide or reduce inbreeding may be highly adaptive for a rodent female at low densities in the increase phase, even at the costs of delaying reproduction.

## Electronic supplementary material

Below is the link to the electronic supplementary material.

Supplementary material 1 (PDF 647 kb)
