# Supplementary material for: The Bruce effect revisited: is pregnancy termination in female rodents an adaptation to ensure breeding success after male turnover in low densities?
Source: Oecologia. 2017 Aug 9;185(1):81–94. doi: 10.1007/s00442-017-3904-6 (PMC5596041; doi:10.1007/s00442-017-3904-6)
Supplement: Supplementary file 1 — Supplementary material 1 (PDF 647 kb) [file 442_2017_3904_MOESM1_ESM.pdf]

Eccard, JA, Ylönen, HY, Dammhahn M 2016: The Bruce effect revisited: is pregnancy termination in female rodents an adaptation to ensure breeding success during low densities?

Appendix 1a: Raw data table of three field experiments on the Bruce effect in female bank voles (*Myodes glareolus*) after the turn-over of males in different compositions of the population. Each line represents one individual female. Experiments differed by composition of males (M) and females (F) (MF, MFFF or MFFF), year and location (G: Potsdam in Germany, F: Konnevesi in Finland) of experiment, and by the cohort of females (a combination of age (YY: young of the year, OW: overwintered, born last year) and reproductive history (n: nulliparous (did not gave birth before the experiment), p: parous). Within each experiment we investigated two turnover treatments after the removal of males from the population: the males were **replaced** by different males , or the same individuals were **returned** as a control. Within the MF experiment we also investigated two spatial scales (enclosure size). In red: replicates that had to be removed from the data analysis because treatment remained incomplete when not all released animals were recaptured from enclosures.

Individual Nr.	Population Name	Composition of Population	Location of Experiment	Year	Month	Enclosure size	Cohort of female	Male turnover	Recapture of male after 1 week	Recapture of male after two weeks	Recapture of female after 2 weeks	Pregnancy of female	Birth of litter (exp. Day)
1	a	MF	G	2015	5	2500	YY-n	returned	0	0	0		
2	b	MF	G	2015	5	2500	YY-n	returned	0	0	0		
3	c	MF	G	2015	5	2500	YY-n	returned	0	0	0		
4	d	MF	G	2015	5	225	YY-n	returned	1	1	1	1	22
5	e	MF	G	2015	5	225	YY-n	returned	1	1	0		
6	f	MF	G	2015	5	225	YY-n	replaced	1	1	1	1	20
7	g	MF	G	2015	5	225	YY-n	replaced	1	1	1	1	26
8	h	MF	G	2015	5	225	YY-n	replaced	1	1	1	0	
9	i	MF	G	2015	5	2500	YY-n	replaced	1	1	0		
10	j	MF	G	2015	5	2500	YY-n	replaced	1	1	0		
11	k	MF	G	2015	6	225	YY-n	returned	0	0	0		
12	l	MF	G	2015	6	225	YY-n	returned	0	0	1	0	
13	m	MF	G	2015	6	2500	YY-n	returned	0	0	1	1	21
14	n	MF	G	2015	6	225	YY-n	replaced	0	0	0		
15	o	MF	G	2015	6	225	YY-n	returned	1	1	1	1	20
16	p	MF	G	2015	6	225	YY-n	returned	1	1	1	1	21
17	q	MF	G	2015	6	2500	YY-n	returned	1	1	1	1	20
18	r	MF	G	2015	6	2500	YY-n	returned	1	1	1	1	22
19	s	MF	G	2015	6	2500	YY-n	replaced	1	0	1	1	22
20	t	MF	G	2015	6	2500	YY-n	replaced	1	1	1	1	27
21	u	MF	G	2015	6	2500	YY-n	replaced	1	1	0		
22	v	MF	G	2015	7	225	YY-n	returned	0	0	0		
23	w	MF	G	2015	7	225	YY-n	returned	0	0	0		
24	x	MF	G	2015	7	225	YY-n	returned	1	1	1	1	26
25	y	MF	G	2015	7	2500	YY-n	returned	1	1	1	1	24
26	z	MF	G	2015	7	2500	YY-n	returned	1	1	1	1	31
27	aa	MF	G	2015	7	2500	YY-n	replaced	1	0	0		
28	ab	MF	G	2015	7	225	YY-n	replaced	1	1	1	0	
29	ac	MF	G	2015	7	225	YY-n	replaced	1	1	1	0	
30	ad	MF	G	2015	7	2500	YY-n	replaced	1	1	1	0	
31	ae	MF	G	2015	7	2500	YY-n	replaced	1	1	0		
32	af	MF	G	2015	7	2500	YY-n	replaced	1	1	1	1	19
33	ag	MF	G	2014	6	225	YY-n	returned	0	0	1	1	23
34	ah	MF	G	2014	6	2500	YY-n	returned	0	0	0		
35	ai	MF	G	2014	6	2500	YY-n	returned	0	0	0		
36	aj	MF	G	2014	6	2500	YY-n	returned	0	1	1	0	
37	aj	MF	G	2014	6	225	YY-n	replaced	1	0	1	1	23
38	ak	MF	G	2014	6	225	YY-n	replaced	1	0	1	1	23
39	al	MF	G	2014	6	225	YY-n	replaced	1	0	1	1	19
40	am	MF	G	2014	6	225	YY-n	replaced	1	0	1	1	23
41	an	MF	G	2014	6	225	YY-n	replaced	1	0	0		

42	ao	MF	G	2014	6	2500	YY-n	replaced	1	0	1	1	30
43	ap	MF	G	2014	6	2500	YY-n	replaced	1	0	1	1	25
44	aq	MF	G	2014	6	2500	YY-n	replaced	1	0	0		
45	ar	MF	G	2014	6	225	YY-n	replaced	1	1	0		
46	as	MF	G	2014	6	2500	YY-n	replaced	1	1	1	1	23
47	at	MF	G	2014	6	2500	YY-n	replaced	1	1	1	0	
48	au	MF	G	2014	6	2500	YY-n	replaced	1	1	0		
49	av	MF	G	2014	7	225	YY-n	returned	0	0	1	1	23
50	aw	MF	G	2014	7	225	YY-n	returned	0	0	1	0	
51	ax	MF	G	2014	7	225	YY-n	returned	0	0	0	0	
52	ay	MF	G	2014	7	225	YY-n	returned	0	0	0		
53	az	MF	G	2014	7	225	YY-n	returned	0	0	0		
54	ba	MF	G	2014	7	2500	YY-n	returned	0	0	1	1	21
55	bb	MF	G	2014	7	2500	YY-n	returned	0	0	0		
56	bc	MF	G	2014	7	2500	YY-n	returned	1	0	0		
57	bd	MF	G	2014	7	225	YY-n	replaced	1	0	1	1	22
58	be	MF	G	2014	7	2500	YY-n	replaced	1	0	1	1	27
59	bf	MF	G	2014	7	225	YY-n	replaced	1	1	1	1	20
60	bg	MF	G	2014	7	225	YY-n	replaced	1	1	1	1	28
61	bh	MF	G	2014	7	225	YY-n	replaced	1	1	1	1	27
62	bi	MF	G	2014	7	2500	YY-n	replaced	1	1	0		
63	bj	MF	G	2014	8	225	YY-n	returned	0	0	1	0	
64	bk	MF	G	2014	8	225	YY-n	returned	0	0	0		
65	cl	MF	G	2014	8	225	YY-n	returned	0	0	0		
66	cm	MF	G	2014	8	225	YY-n	returned	0	0	0		
67	cn	MF	G	2014	8	2500	YY-n	returned	0	0	0		
68	co	MF	G	2014	8	225	YY-n	returned	1	1	1	1	21
69	cp	MF	G	2014	8	2500	YY-n	returned	1	1	0		
70	cq	MF	G	2014	8	2500	YY-n	returned	1	1	1	1	19
71	cr	MF	G	2014	8	225	YY-n	replaced	1	0	0		
72	cs	MF	G	2014	8	2500	YY-n	replaced	1	0	1	1	22
73	ct	MF	G	2014	8	225	YY-n	replaced	1	1	1	1	20
74	cu	MF	G	2014	8	2500	YY-n	replaced	1	1	1	1	24
75	25a	MFFF	F	2000	5	2500	OW-n	replaced	1	1	1	0	
76	25a	MFFF	F	2000	5	2500	OW-n	replaced	1	1	1	0	
77	25b	MFFF	F	2000	5	2500	OW-n	replaced	1	1	1	1	20
78	25b	MFFF	F	2000	5	2500	OW-n	replaced	1	1	1	1	21
79	25b	MFFF	F	2000	5	2500	OW-n	replaced	1	1	1	0	
80	25c	MFFF	F	2000	5	2500	OW-n	replaced	1	1	1	1	20
81	25c	MFFF	F	2000	5	2500	OW-n	replaced	1	1	1	1	21
82	25c	MFFF	F	2000	5	2500	OW-n	replaced	1	1	0		
83	25a	MFFF	F	2000	5	2500	OW-n	replaced	1	1	1	1	21
84	25d	MFFF	F	2000	5	2500	OW-n	replaced	0	1	1	1	21
85	25d	MFFF	F	2000	5	2500	OW-n	replaced	0	1	0		
86	25d	MFFF	F	2000	5	2500	OW-n	replaced	0	1	1	1	21
87	222	MFFF	F	2000	6	2500	YY-n	replaced	1	1	1	1	20
88	222	MFFF	F	2000	6	2500	YY-n	replaced	1	1	1	1	20
89	222	MFFF	F	2000	6	2500	YY-n	replaced	1	1	1	1	21
90	223	MFFF	F	2000	6	2500	YY-n	returned	1	1	1	1	20
91	223	MFFF	F	2000	6	2500	YY-n	returned	1	1	1	1	20
92	223	MFFF	F	2000	6	2500	YY-n	returned	1	1	1	1	23
93	224	MFFF	F	2000	6	2500	YY-n	replaced	1	1	1	1	18
94	224	MFFF	F	2000	6	2500	YY-n	replaced	1	1	1	1	18
95	224	MFFF	F	2000	6	2500	YY-n	replaced	1	1	1	1	20
96	235	MFFF	F	2000	6	2500	YY-n	replaced	1	1	1	1	23
97	235	MFFF	F	2000	6	2500	YY-n	replaced	1	1	1	1	26
98	235	MFFF	F	2000	6	2500	YY-n	replaced	1	1	0		
99	236	MFFF	F	2000	6	2500	YY-n	replaced	1	1	1	1	23
100	236	MFFF	F	2000	6	2500	YY-n	replaced	1	1	0		
101	236	MFFF	F	2000	6	2500	YY-n	replaced	1	1	1	0	
102	237	MFFF	F	2000	6	2500	YY-n	returned	1	1	1	1	21
103	237	MFFF	F	2000	6	2500	YY-n	returned	1	1	1	1	23
104	237	MFFF	F	2000	6	2500	YY-n	returned	1	1	1	1	23
105	238	MFFF	F	2000	6	2500	YY-n	returned	1	1	1	1	20
106	238	MFFF	F	2000	6	2500	YY-n	returned	1	1	1	0	
107	238	MFFF	F	2000	6	2500	YY-n	returned	1	1	1	0	
108	141	MFFF	F	1999	7b	2500	YY-n	returned	1	1	1	1	18
109	141	MFFF	F	1999	7b	2500	YY-p	returned	1	1	1	1	31
110	141	MFFF	F	1999	7b	2500	YY-p	returned	1	1	1	1	22
111	142	MFFF	F	1999	7b	2500	YY-p	returned	1	1	0		
112	142	MFFF	F	1999	7b	2500	OW-p	returned	1	1	1	1	21
113	142	MFFF	F	1999	7b	2500	YY-n	returned	1	1	1	1	20
114	143	MFFF	F	1999	7b	2500	OW-p	replaced	1	1	1	1	18

115	143	MFFF	F	1999	7b	2500	OW-n	replaced	1	1	1	1	21
116	143	MFFF	F	1999	7b	2500	OW-p	replaced	1	1	1	1	20
117	148	MFFF	F	1999	7b	2500	OW-p	replaced	1	1	0		
118	148	MFFF	F	1999	7b	2500	YY-n	replaced	1	1	1	0	
119	148	MFFF	F	1999	7b	2500	OW-p	replaced	1	1	1	1	22
120	121	MMMFFF	F	1999	6	2500	OW-p	returned	1	1	1	1	20
121	121	MMMFFF	F	1999	6	2500	OW-p	returned	1	1	1	1	20
122	121	MMMFFF	F	1999	6	2500	YY-n	returned	1	1	1	1	22
123	128	MMMFFF	F	1999	6	2500	YY-n	replaced	1	1	1	1	20
124	128	MMMFFF	F	1999	6	2500	YY-p	replaced	1	1	1	1	20
125	128	MMMFFF	F	1999	6	2500	YY-p	replaced	1	1	1	1	22
126	131	MMMFFF	F	1999	7a	2500	OW-n	returned	1	1	1	0	
127	131	MMMFFF	F	1999	7a	2500	OW-p	returned	1	1	1	1	26
128	131	MMMFFF	F	1999	7a	2500	YY-p	returned	1	1	1	1	27
129	132	MMMFFF	F	1999	7a	2500	YY-n	replaced	1	1	0		
130	132	MMMFFF	F	1999	7a	2500	YY-p	replaced	1	1	1	1	25
131	132	MMMFFF	F	1999	7a	2500	YY-n	replaced	1	1	1	1	19
132	133	MMMFFF	F	1999	7a	2500	YY-n	returned	1	1	1	0	
133	133	MMMFFF	F	1999	7a	2500	?-p	returned	1	1	1	1	19
134	133	MMMFFF	F	1999	7a	2500	OW-p	returned	1	1	1	1	21
135	138	MMMFFF	F	1999	7a	2500	OW-p	replaced	1	1	1	0	
136	138	MMMFFF	F	1999	7a	2500	OW-p	replaced	1	1	1	1	20
137	138	MMMFFF	F	1999	7a	2500	YY-n	replaced	1	1	1	1	21

Appendix 1b: Raw data table of laboratory experiment on the Bruce effect in female bank voles (*Myodes glareolus*) after the turn-over of the paired males. We investigated two turnover treatments after the removal of the male: the male was either **replaced** by a different male, or the same individual was **returned** as a control. Females differed by their cohort (a combination of age (YY: young of the year, OW: overwintered, born last year) and reproductive history (n: nulliparous (did not gave birth before the experiment), p: parous). The experiment was conducted in a breeding colony in Konnevesi, Finland kiept indoors in standard breeding cages in 1999.

**Individual Nr.**	**Female age**	**Female reproductive history**	**Cohort**	**Male turnover treatment**	**Gravid?**	**Birth of litter (experimental day)**
1	ow	n	OW-n	returned	0	NA
2	ow	n	OW-n	returned	0	NA
3	ow	n	OW-n	returned	1	24
4	ow	n	OW-n	returned	0	NA
5	ow	n	OW-n	returned	1	20
6	ow	n	OW-n	returned	0	NA
7	ow	n	OW-n	returned	1	20
8	ow	n	OW-n	returned	1	20
9	ow	n	OW-n	returned	1	22
10	ow	n	OW-n	returned	1	20
11	ow	n	OW-n	returned	0	NA
12	ow	n	OW-n	returned	1	20
13	ow	n	OW-n	returned	1	23
14	ow	n	OW-n	returned	1	22
15	ow	n	OW-n	returned	0	NA
16	ow	n	OW-n	returned	0	NA
17	ow	n	OW-n	returned	1	29
18	ow	n	OW-n	returned	1	31
19	ow	n	OW-n	returned	1	20
20	ow	n	OW-n	returned	1	20
21	ow	n	OW-n	returned	1	22
22	ow	n	OW-n	returned	0	NA
23	ow	n	OW-n	returned	0	NA
24	ow	n	OW-n	returned	0	NA
25	ow	n	OW-n	returned	1	25

26	ow	n	OW-n	returned	0	NA
27	ow	n	OW-n	returned	1	30
28	ow	n	OW-n	returned	1	19
29	ow	n	OW-n	returned	0	NA
30	ow	n	OW-n	returned	1	28
31	ow	n	OW-n	returned	0	NA
32	ow	n	OW-n	returned	0	NA
33	ow	n	OW-n	returned	0	NA
34	ow	n	OW-n	returned	0	NA
35	ow	n	OW-n	returned	1	21
36	ow	n	OW-n	returned	1	19
37	ow	n	OW-n	returned	0	NA
38	ow	n	OW-n	returned	1	26
39	ow	n	OW-n	returned	1	18
40	ow	n	OW-n	returned	0	NA
41	ow	n	OW-n	returned	1	18
42	ow	n	OW-n	returned	0	NA
43	ow	n	OW-n	returned	1	20
44	ow	n	OW-n	returned	0	NA
45	ow	n	OW-n	returned	0	NA
46	ow	n	OW-n	returned	1	20
47	ow	n	OW-n	returned	1	18
48	ow	n	OW-n	returned	0	NA
49	ow	n	OW-n	returned	1	19
50	ow	n	OW-n	returned	1	19
51	ow	n	OW-n	returned	1	20
52	yy	n	YY-n	returned	1	19
53	yy	n	YY-n	returned	1	18
54	yy	n	YY-n	returned	1	19
55	yy	n	YY-n	returned	1	19
56	yy	n	YY-n	returned	1	23
57	yy	n	YY-n	returned	1	25
58	yy	n	YY-n	returned	1	29
59	yy	n	YY-n	returned	1	27
60	yy	n	YY-n	returned	0	NA
61	yy	n	YY-n	returned	1	21
62	yy	n	YY-n	returned	1	23
63	yy	n	YY-n	returned	1	20
64	yy	n	YY-n	returned	1	19
65	yy	n	YY-n	returned	1	19
66	yy	n	YY-n	returned	0	NA
67	yy	n	YY-n	returned	0	NA
68	yy	n	YY-n	returned	1	19
69	yy	n	YY-n	returned	0	NA
70	yy	n	YY-n	returned	1	32
73	yy	p	YY-p	returned	1	20
74	yy	p	YY-p	returned	1	25
75	yy	p	YY-p	returned	0	NA
76	yy	p	YY-p	returned	1	20
77	yy	p	YY-p	returned	1	20
78	yy	p	YY-p	returned	0	NA
79	yy	p	YY-p	returned	1	18
80	yy	p	YY-p	returned	1	20
81	yy	p	YY-p	returned	1	22
82	yy	p	YY-p	returned	1	21
83	yy	p	YY-p	returned	0	NA
84	yy	p	YY-p	returned	1	17
85	yy	p	YY-p	returned	1	19
86	yy	p	YY-p	returned	1	25
87	yy	p	YY-p	returned	1	19
88	yy	p	YY-p	returned	1	18
89	yy	p	YY-p	returned	0	NA
90	yy	p	YY-p	returned	1	22
91	yy	p	YY-p	replaced	1	21
92	yy	p	YY-p	replaced	1	19
93	ow	n	OW-n	replaced	1	28
94	ow	n	OW-n	replaced	0	NA
95	ow	n	OW-n	replaced	1	20
96	ow	n	OW-n	replaced	1	18
97	ow	n	OW-n	replaced	1	25
98	ow	n	OW-n	replaced	0	NA
99	ow	n	OW-n	replaced	1	28
100	ow	n	OW-n	replaced	1	27

101	ow	n	OW-n	replaced	1	28
102	ow	n	OW-n	replaced	1	27
103	ow	n	OW-n	replaced	1	20
104	ow	n	OW-n	replaced	1	27
105	ow	n	OW-n	replaced	0	NA
106	ow	n	OW-n	replaced	1	20
107	ow	n	OW-n	replaced	1	20
108	ow	n	OW-n	replaced	0	NA
109	ow	n	OW-n	replaced	1	23
110	ow	n	OW-n	replaced	1	20
111	ow	n	OW-n	replaced	1	20
112	ow	n	OW-n	replaced	0	NA
113	ow	n	OW-n	replaced	1	20
114	ow	n	OW-n	replaced	0	NA
115	ow	n	OW-n	replaced	1	27
116	ow	n	OW-n	replaced	1	26
117	ow	n	OW-n	replaced	0	NA
118	ow	n	OW-n	replaced	0	NA
119	ow	n	OW-n	replaced	0	NA
120	ow	n	OW-n	replaced	1	26
121	ow	n	OW-n	replaced	1	26
122	ow	n	OW-n	replaced	1	26
123	ow	n	OW-n	replaced	1	19
124	ow	n	OW-n	replaced	1	19
125	ow	n	OW-n	replaced	1	26
126	ow	n	OW-n	replaced	0	NA
127	ow	n	OW-n	replaced	0	NA
128	ow	n	OW-n	replaced	0	NA
129	ow	n	OW-n	replaced	1	18
130	ow	n	OW-n	replaced	1	19
131	ow	n	OW-n	replaced	0	NA
132	ow	n	OW-n	replaced	1	29
133	ow	n	OW-n	replaced	1	19
134	ow	n	OW-n	replaced	1	19
135	ow	n	OW-n	replaced	0	NA
136	ow	n	OW-n	replaced	1	28
137	ow	n	OW-n	replaced	0	NA
138	ow	n	OW-n	replaced	1	29
139	ow	n	OW-n	replaced	1	18
140	ow	n	OW-n	replaced	0	NA
141	ow	n	OW-n	replaced	1	23
142	ow	n	OW-n	replaced	0	NA
143	ow	n	OW-n	replaced	1	31
144	ow	n	OW-n	replaced	1	20
145	ow	n	OW-n	replaced	1	20
146	ow	n	OW-n	replaced	0	NA
147	ow	n	OW-n	replaced	1	26
148	ow	n	OW-n	replaced	1	19
149	ow	n	OW-n	replaced	0	NA
150	ow	n	OW-n	replaced	0	NA
151	ow	n	OW-n	replaced	1	19
152	ow	n	OW-n	replaced	1	18
153	ow	n	OW-n	replaced	0	NA
154	ow	n	OW-n	replaced	0	NA
155	yy	n	YY-n	replaced	1	20
156	yy	n	YY-n	replaced	1	27
157	yy	n	YY-n	replaced	1	23
158	yy	n	YY-n	replaced	1	30
159	yy	n	YY-n	replaced	1	33
160	yy	n	YY-n	replaced	1	28
161	yy	n	YY-n	replaced	1	23
162	yy	n	YY-n	replaced	1	22
163	yy	n	YY-n	replaced	0	NA
164	yy	n	YY-n	replaced	1	25
165	yy	n	YY-n	replaced	1	24
166	yy	n	YY-n	replaced	1	29
167	yy	n	YY-n	replaced	1	20
168	yy	n	YY-n	replaced	1	25
169	yy	n	YY-n	replaced	1	28
170	yy	n	YY-n	replaced	1	31
171	yy	n	YY-n	replaced	1	21
172	yy	n	YY-n	replaced	1	18
173	yy	n	YY-n	replaced	1	26

174	yy	n	YY-n	replaced	1	25
175	yy	n	YY-n	replaced	1	30
176	yy	n	YY-n	replaced	1	22
177	yy	n	YY-n	replaced	1	19
178	yy	n	YY-n	replaced	1	21
179	yy	n	YY-n	replaced	1	24
180	yy	n	YY-n	replaced	1	22
181	yy	n	YY-n	replaced	1	20
182	yy	n	YY-n	replaced	1	21
183	yy	n	YY-n	replaced	1	28
184	yy	n	YY-n	replaced	1	19
185	yy	n	YY-n	replaced	0	NA
186	yy	n	YY-n	replaced	0	NA
187	yy	n	YY-n	replaced	0	NA
188	yy	n	YY-n	replaced	1	20
189	yy	n	YY-n	replaced	1	22
190	yy	n	YY-n	replaced	0	NA
191	yy	n	YY-n	replaced	0	NA
192	yy	n	YY-n	replaced	1	20
193	yy	n	YY-n	replaced	1	32
194	yy	n	YY-n	replaced	1	28
197	yy	p	YY-p	replaced	1	19
198	yy	p	YY-p	replaced	1	19
199	yy	p	YY-p	replaced	1	20
200	yy	p	YY-p	replaced	1	22
201	yy	p	YY-p	replaced	1	19
202	yy	p	YY-p	replaced	1	20
203	yy	p	YY-p	replaced	1	19
204	yy	p	YY-p	replaced	1	19
205	yy	p	YY-p	replaced	1	21
206	yy	p	YY-p	replaced	1	22
207	yy	p	YY-p	replaced	1	21
208	yy	p	YY-p	replaced	1	27
